# School meal provision, health, and cognitive function in a Nordic setting – the ProMeal-study: description of methodology and the Nordic context

**DOI:** 10.3402/fnr.v60.30468

**Published:** 2016-08-10

**Authors:** Maria Waling, Anna S. Olafsdottir, Hanna Lagström, Hege Wergedahl, Bert Jonsson, Cecilia Olsson, Eldbjørg Fossgard, Asle Holthe, Sanna Talvia, Ingibjorg Gunnarsdottir, Agneta Hörnell

**Affiliations:** 1 Department of Food and Nutrition, Umeå University, Umeå, Sweden; 2 School of Education, University of Iceland, Reykjavik, Iceland; 3 Turku Institute of Child and Youth Research, University of Turku, Turku, Finland; 4 Faculty of Education, Bergen University College, Bergen, Norway; 5 Department of Psychology, Umeå University, Umeå, Sweden; 6 Unit for Nutrition Research, Landspitali - The National University Hospital of Iceland and Faculty of Food Science and Nutrition, School of Health Sciences, University of Iceland, Reykjavík, Iceland

**Keywords:** school meals, Nordic countries, education, cognitive function, dietary intake

## Abstract

**Background:**

School meals, if both nutritious and attractive, provide a unique opportunity to improve health equality and public health.

**Objective:**

To describe the study rationale, data collection, and background of participants in the study ‘Prospects for promoting health and performance by school meals in Nordic countries’ (ProMeal). The general aim was to determine whether overall healthiness of the diet and learning conditions in children can be improved by school lunches, and to capture the main concerns regarding school lunches among children in a Nordic context.

**Design:**

A cross-sectional, multidisciplinary study was performed in Finland, Iceland, Norway, and Sweden on pupils (*n*=837) born in 2003.

**Results:**

In total 3,928 pictures of school lunches were taken to capture pupils’ school lunch intake. A mean of 85% of all parents responded to a questionnaire about socioeconomic background, dietary intake, and habitual physical activity at home. Cognitive function was measured on one occasion on 93% of the pupils during optimal conditions with a Stroop and a Child Operation Span test. A mean of 169 pupils also did an Integrated Visual and Auditory Continuous Performance Test after lunch over 3 days. In total, 37,413 10-sec observations of classroom learning behavior were performed. In addition, 753 empathy-based stories were written and 78 focus groups were conducted. The pupils had high socioeconomic status.

**Conclusions:**

This study will give new insights into which future interventions are needed to improve pupils’ school lunch intake and learning. The study will provide valuable information for policy making, not least in countries where the history of school meals is shorter than in some of the Nordic countries.

School meals, if both nutritious and attractive, provide a unique opportunity to improve health equality and public health (1). Despite this, only a few studies have been conducted to evaluate the effect of both school meals and the organization of these meals on total dietary intake (2), classroom learning behavior (3, 4), and cognitive function (5, 6).

The Nordic countries have common dietary recommendations (7), but there are differences in the implementation of guidelines for municipalities and others responsible for organizing school lunches. Furthermore, there are important differences in legislation related to the organization of school lunches in the Nordic countries (8). Finland and Sweden are two of the few countries in the world with a legislation that makes school meals free of charge. In Finland, it is further legislated that school meals should be ‘well-balanced’, and in Sweden ‘nutritious’. The school meal organizations of the other Nordic countries range from giving parents/caregivers total economic and practical responsibility to government subsidizing at varying levels.

The Nordic countries’ similar dietary habits but different ways of organizing school lunches give a unique opportunity to study the effect of different ways of school meal organization on pupils’ total dietary intake, classroom learning behavior, and cognitive function. The overall aim of the study, ‘Prospects for promoting health and performance by school meals in Nordic countries’ (ProMeal), was to determine whether the overall healthiness of the diet and learning conditions in children can be improved by school lunches, and to capture the main concerns regarding school lunches among children in a Nordic context. More specific aims were to study the relationship between school lunch intake and overall healthiness of the diet, the effect of school lunches on cognitive function and classroom learning behavior, as well as the school meal environment and pupils’ perspectives of school lunches. The present paper describes the study rationale, design, data collection, and participating pupils in the ProMeal-study.

## Methods

### School meal organization

Among the four Nordic countries included in this study, three different school meal organization systems were represented.

#### Finland

Dating back to a law from 1943 (9), all Finnish school children in compulsory school (aged 7–15), should be provided with a hot, well-balanced school lunch free of charge (10). In 2008, the National Nutrition Council published the so called Finnish School Lunch Recommendations, based on the Finnish Nutrition Recommendations (11), which in turn are based on the Nordic Nutrition Recommendations (7). The Finnish School Lunch Recommendations describe the requirements of food quality and how often various food items should be served. Education acts and decrees, along with national core curricula, local curricula, and school-level curricula, are central documents governing school lunches. Municipalities and other education providers are responsible for resources and the practical implementation of school meals. A community unit, a public utility, or a private company is responsible for planning, preparing, and serving the meals. These units are responsible for ensuring that nutritious meals are served by self-regulation, but there is no governmental monitoring of the school meals in Finland. A typical Finnish school lunch includes a hot meal, vegetables and/or fruit, bread, a spreadable fat, and a drink (water or milk).

#### Iceland

In 1995, it was stated in the Icelandic Educational Act that school meals should be provided at school time. It is most common for the cost to be covered partly by the pupils’ families and partly by the municipalities. In the revised version of the act published in 2008 (12), it was stated that the nutrient composition of school meals should meet the Icelandic nutrient and food-based recommendations (13). These recommendations are based on the Nordic Nutrition Recommendations (7). There is no official or governmental monitoring of the school meals, but the schools are expected to follow the recommendations. Prior to 1995, all pupils brought a packed lunch, and some pupils still bring their own lunches to school. In some schools, private companies are responsible for the planning, preparing, and serving of school meals, while other schools provide the meals themselves. In both cases, the individual schools are always responsible for the eating environment, including providing an eating space, as well as organizing the pupil flow and eating. A typical Icelandic school lunch includes a warm meal and water as a drink. Fruit and/or vegetables are expected to be a part of each school meal, but serving methods are diverse. By default, fruit and/or vegetables are self-served or provided at a salad bar. Unlike Sweden and Finland, bread is only served occasionally as a part of Icelandic school lunches.

#### Norway

In Norway, pupils usually bring their own packed lunch to school. There is no national legislation for school meal provision, but in 2003, guidelines were published for healthy school meals in primary and secondary schools (aged 6–18) (14). The guidelines are built on the concept that children bring their own lunch boxes and state that schools should offer sufficient time for eating, supervision during school meals, a pleasant eating environment, and a maximum of 3 to 4 h between meals.

The pupils often eat their lunch in the class room. Schools have been offered a government-subsided milk subscription since the late 1960s, allowing 3 dl of milk to be served daily, and a fruit and vegetable subscription from the 1990s. Beginning in 2009, it was statutory to offer pupils at lower secondary and combined primary/lower secondary schools (aged 6–15) a free piece of fruit or vegetable daily, but this requirement was eliminated by the new government, in 2014. A typical Norwegian lunch box includes sandwiches of bread or crispbread with a spreadable fat, ham, salted meat, cheese, liver paté or a sweet spread with fruit and some vegetables, and milk or water to drink. Since there is no legislation regarding school meals, there is no monitoring of them in Norway.

#### Sweden

The foundation of the Swedish school meal system was laid during the 1940s, and since the early 1970s, free school meals have been served to all pupils in compulsory school (pupils aged 7 to 15). In 1997, the Swedish Educational Act was instituted with the requirement that school meals should be free of charge in all schools. In 2011, an addition to the act required that the meal should be nutritious, that is, corresponding to the Swedish nutrition recommendations, which were based on Nordic Nutrition Recommendations (7), (15). The meals are government funded, but the responsibility for serving school meals lies with municipalities for municipality schools and company owners for independent schools. A local municipality unit is usually responsible for planning, preparing, and serving the school lunches in the municipality schools, while the school is responsible for the eating environment (pupils usually eat in a special school restaurant within the school), timing, and the teachers’ roles during meal time. The Swedish Schools Inspectorate is responsible for ensuring that schools have routines and work systematically to serve nutritious meals. A typical Swedish school lunch includes a hot meal, a choice of four to six different kinds of vegetables or salads (sometimes fruit is served), crispbread, a spreadable fat, and a drink (water or milk).

### Study design

ProMeal is a cross-sectional study with a multidisciplinary approach in which data were collected between October 2013 and May 2014.

### Recruitment

We aimed to recruit children born in 2003, that is, children in grade 4 in Finland and Sweden, and grade 5 in Iceland and Norway. Some children included in the classes were born 1 year earlier (*n*=11) or 1 year later than 2003 (*n*=4). Schools were recruited at each study site with the goal of including schools from areas with diverse socioeconomic and ethnic characteristics. The recruitment started during spring 2013 and continued in parallel to the data collection until spring 2014. In Sweden, school leaders, and after their acceptance teachers, were contacted directly and informed about the study. In Finland, Iceland, and Norway, the municipalities were contacted first for consent to contact school leaders and, once the school leaders accepted, the teachers were contacted. Information letters and informed consent forms were sent home to parents/caregivers and pupils to consider participation.

A power calculation showed that including 200 children in each country would enable detection of differences in cognitive functions and classroom learning behavior related to dietary intake with a power of 80% and significance level of *P*<0.05.

### Data collection

**Fig. 1:**
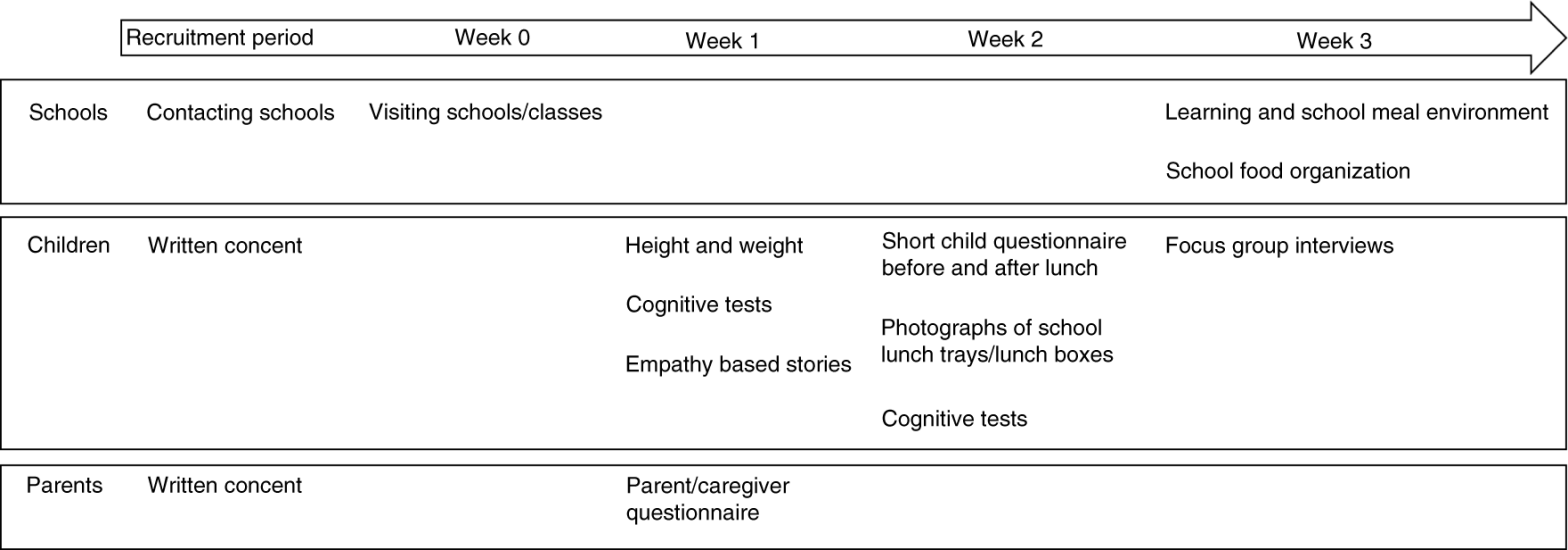
Study design and measurement overview of the ProMeal-study.

An overview of the data collection methods can be found in Fig. 1. In each class, data were collected during a 3-week period. During the first and the third week, the class was visited on 1 day, and during the second week, the class was visited on 5 days. Data were collected with the same instruments and in the same structured way in all countries to facilitate comparisons. Sometimes protocols were slightly modified to better fit country-specific circumstances. Protocols were tested in a Swedish school during a workshop, before the data collection began. Researchers who were involved in data collection tested and discussed the different methods to form a common view of different aspects of data collection. During the data collection process, regular web-based meetings were held and issues that came up were discussed.

#### Anthropometric and socioeconomic data

Pupils’ body weight and height was measured by a trained researcher in each country. Body weight was weighed to the nearest kilogram (with one decimal) in light clothing and without shoes with an electronic scale (Seca Robusta 813, USA). Height was measured to the nearest millimeter with a portable wall stadiometer (Marsden HM-250P, United Kingdom). Pupils were classified as normal weight, overweight, or obese using the International Obesity Task Force (IOTF) definition (16).

Socioeconomic and background data were collected through a parent/caregiver questionnaire. In Iceland, Norway, and Sweden, the questionnaire was sent to parents/caregivers through an e-mail, which was filled out online. Parents/caregivers who did not respond to the questionnaire inquiry were reminded. In Finland, the questionnaire was only administrated through a telephone interview. The questionnaire covered aspects regarding education and occupation of parents/caregivers, ethnicity, family constellation, rating of the child's health, and the child's diseases/health conditions, if any. In Norway, the questionnaire did not include questions about ethnicity, the education of the parent/caregiver not filling out the questionnaire, the parent's/caregivers’ occupations, who the child lived with, or a rating of the child's health. These questions were added to the questionnaire after the application for ethical approval was approved in Norway and there was no time to revise the previously approved application. The total parent/caregiver questionnaire took about 15–20 min to fill out.

#### Dietary intake and physical activity level outside school

Dietary habits at home were assessed through a quantitative food frequency questionnaire (FFQ), which was developed and validated as part of the Nordic Monitoring project (17, 18). This FFQ was developed to monitor the intake of selected food items and food groups (20 questions), known to contribute to the total healthiness of the diet in the Nordic countries. The questions concerned the frequency of intake of commonly consumed foods that contribute substantially to the total intake of fat, saturated fatty acids, sucrose, and dietary fiber in the Nordic diet. The questions covered usual consumption during the past 12 months and were answered by the parents/caregivers as part of the parent/caregiver questionnaire described above. The questionnaire had already been translated into the different Nordic languages.

Daily dietary intake from school lunch was assessed through a photographic method (19, 20), that was validated as part of the ProMeal-study (unpublished observation). During study week two, all school lunch trays/boxes (including extra helpings and leftovers) were photographed by researchers and trained assistants from two angles; from above and at 45 degrees. A half- and full-weighed reference portion was photographed and used for comparison with amounts taken, any additional helpings, and leftovers in Finland, Iceland, and Sweden. These were then used for estimating the total amount eaten and, most importantly to be used in future analysis to define a very low intake (less than one-half of the meal provided). In Norway, standard portions from a nutritional calculation program were used to assess amounts. On a few occasions, the pupils forgot to photograph extra helpings or leftovers, and if no notes had been taken (e.g. ‘missed leftover photograph, but all food eaten’), that day was noted as ‘missing’. The following nutritional calculation programs and food databases were used in the participating countries: in Finland, AivoDiet 2.0.2.3 and Fineli^®^ – the Finnish Food Composition Database; in Iceland, Icefood 2.0 and the National Nutrition Database, ISGEM; in Norway, Kostholdsplanleggeren and the Norwegian food composition database from 2014 (both from the Norwegian Directorate of health and the Norwegian Food Safety Authority); and in Sweden, Dietist Net Pro version 15.02.14 and the National Food Agency's Nutrition Database 15.12.13.

The pupils also filled out two short structured questionnaires daily during study week two; one before and one after lunch. In both questionnaires, they were instructed to rate how hungry they were on a visual analogue scale (VAS), which is a method that has been used in earlier studies for similar ratings of satiety and appetite (21, 22). After lunch, they were further instructed to rate both the lunch meal and how they felt on a VAS. If they did not finish their meals, they were asked to give the main reason for the same. Before lunch, they were also asked if and what they had eaten for breakfast, as well as snacks between breakfast and lunch. The questionnaire also included a question about when they went to bed the evening before and when they got up in the morning of the present day.

Physical activity level at home was assessed through four questions as part of the parental/caregiver questionnaire in Finland, Iceland, and Sweden. The aim was not to capture the total physical activity of each individual, but rather to get a general estimate of the pupil's physical activity level outside school for use as a co-variate in future analyses. One question concerned daily sedentary behavior, that is, how many hours per day the pupils spent using a computer, tablet, or smartphone, as well as playing video games and watching television. Two questions were regarding physical activity and were divided into moderate (activities in which one gets slightly out of breath, for example, dancing, horseback riding, or line skating) and strenuous (activities in which one gets out of breath and sweaty, for example, activities like running, ball games, and cross-country skiing). Five predefined frequencies were given (never, 1–2 times per week, 3–4 times per week, 5–6 times per week, or all days of the week). The fourth question was regarding how many times per week the child walked or biked to school and back home.

#### Children's perspective of school lunches

Empathy-based stories (23) were used to study pupils’ experiences and perspectives of school meal situations. Pupils were randomly selected to write short stories based on one of two different frame stories. The frame story helped direct the child to write a story about a school lunch that was either a bad or a good experience. Pupils could write about something that they had experienced themselves, been told, or an imaginary but still possible story.

Further, focus groups were conducted to study children's understandings and way of discussing school lunches (24). The purpose of the focus groups was to investigate and compare experiences and beliefs about the school meals. A structured topic guide was used and the interviews were monitored by one moderator and one assistant. A selection of photos of various dishes, meals, and school lunch contexts were shown in order to stimulate the pupils to talk. The interviews were digitally recorded and transcribed verbatim. In Iceland and Sweden, all groups were separated by gender; in Norway, most groups (16 of 25) were separated by gender; and in Finland, most groups (13 of 19) were mixed gender.

#### Cognitive function and classroom learning behavior

Measures of cognitive functioning were conducted during study week one and two. Cognitive functioning in the present project is defined as working memory (WM) capacity, the ability to inhibit a prepotent response, speed of information processing, and attention and self-control. In the first study week, a complex WM task (25) and a computerized Stroop test (26) was administered. Operation span (27) is frequently denoted as one of the most reliable and valid instruments of WM (28). In the present study, a child version was used, which in a previous study was found to have high internal consistency with other measures of WM capacity, digit span, and block span (25). There are a number of versions of Stroop task tests available that differ slightly, but the overall picture is that the Stroop effects are a robust phenomenon (29). Analysis of test–re-test reliability have found that computerized Stroop tests have higher reliability than the conventional test (30). These tests were performed with all participating pupils during an early lesson in order to get a reference value of cognitive function in conditions as ‘optimal’ as possible. In the complex WM task, denoted as the Child Operation span (CO-span), the children were required to solve mathematical tasks while retaining letters in their short-term memory. The number of letters retained was used as measure of WM capacity. In the Stroop task, the participants were shown a sequence of words in the national language and were required to indicate the color of the letters that either was incongruent (the word ‘red’ printed in ‘blue’), congruent (the word ‘red’ printed in ‘red’), or neutral (the word presented in color with no relation to the semantic meaning). Reaction times to incongruent and congruent conditions were used as measures of cognitive inhibition and processing speed, respectively.

The second study week, the Integrated Visual and Auditory Continuous Performance Test (IVA+Plus) (31), which measures a person's ability to concentrate and remain focused, was performed on a random sub-sample of five pupils in each class during 3 days, 1 to 2 h after lunch. In Sweden, the CO-span and Stroop tests were also performed on an additional five randomized pupils on the same 3 days as the IVA+Plus test was performed. The IVA+Plus test is a standardized instrument with normative data based on 1,700 individuals. The practice effects for IVA+Plus are generally quite small (32).

In addition to the computer tests, structured observations were performed by researchers and trained research assistants to study learning-related classroom behavior in the first lesson after lunch, during study week two. The observation schedule was based on the procedure by Blatchford et al. (33, 34) and comprised categories for time spent in different social settings (child-teacher interaction, child-child interaction, or individual behavior) and in different work settings (individual, group, or whole class). Within the social settings, there were mutually exclusive categories to describe how children behaved (work, social, off-task). The goal was to capture whether or not the child was on-task (concentrated on the task) or off-task (disengaged, disrupted).

#### School meal and learning environment

The school meal environment was systematically observed in Finland, Iceland, and Sweden with a standardized protocol constructed by the research group. The first area observed was the physical eating environment, for example, size of room, noise-protective materials, temperature, light, art, tables, chairs, material of plates, and cups. Other observations were the number of children eating at the same time, queueing, written rules, staff eating with the pupils, serving practices, commercial messages, and the accessibility of the school menu for pupils and parents. In Norway, an adapted protocol was used since pupils there eat in the classroom.

Physical learning environment was also studied through observations of the class rooms; for example, the size of the classroom, how pupils were seated, the placement of the teacher, teaching-related equipment, extra work stations, art, light, noise-protective materials, and written rules.

#### School food organization

Different aspects of school food organization were evaluated in all countries with a validated instrument called School Food Sweden (35). The instrument consists of three levels/areas: 1) Variety, nutrition, and food safety; 2) Service, pedagogical thinking, and environmental impact; and 3) Organization and management. The full instrument was only used in Sweden since it was country-specific. Adapted versions of levels 2 and 3 were used in Finland, Iceland, and Norway. In Sweden, the head food service personnel at each school answered the original developed web-based tool. In Finland, the questionnaires were answered by the researchers after observations and conversations with food service personnel. In Iceland, a translated paper questionnaire was answered by food service personnel. In Norway, parts of the questionnaire in level 3 was used, and modified to better fit Norwegian circumstances.

### Statistical analysis

In the analysis presented in the present paper, the Chi-square test for independence was used to test potential differences in distribution between categorical variables in the parent/caregiver questionnaire. For variables with cells with an expected count less than five, the Fisher's exact test was used. Descriptive data are presented as mean and standard deviation. For continuous variables, a one-way between-groups analysis of variance was used. If statistically significant differences were seen, a *post-hoc* test (Tukey's HSD) was used to show between which groups the difference occurred. Eta squared was calculated to estimate effect size, and Cohen's classification was used for interpretation of the effect size: 0.01=small effect; 0.06=medium effect; and 0.14=large effect. Results were considered statistically significant if the *P*-value was <0.05. IBM SPSS Statistics, version 22.0, was used (Armonk, NY: IBM Corp.) for all statistical analysis.

This study was conducted according to the guidelines laid down in the Helsinki Declaration of 1975, as revised in 2008, and all procedures involving human subjects were approved by the Ethical Committee of the University of Turku in Finland, The National Bioethics Committee (56363); The Icelandic Data Protection Authority (VSN-13-088) in Iceland; The Data Protection Official for Research in Norway; and The Regional Research Ethics Review Board, the Faculty of Medicine, Umeå University, in Sweden (2013-212-31Ö). Written informed consent was obtained from all parents/caregivers in the participating countries. In Finland, informed consent was also collected from the pupils. In all countries, the pupils were able to deny participation even if parents/caregivers had consented participation.

## Results

**Fig. 2:**
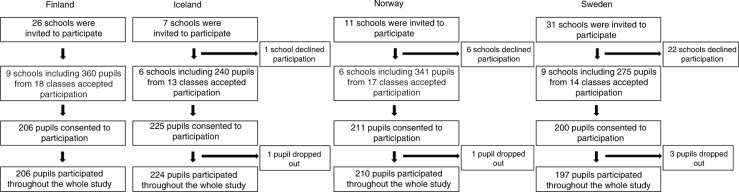
Flow-cart of participants in Finland, Iceland, Norway, and Sweden in the ProMeal-study.

In total, 75 schools were invited to participate in the study (variation between countries 7–31) and 30 schools accepted the invitation (variation between countries 6–9). For country-specific numbers, please see Fig. 2. The schools that accepted the invitation had a total of 1,216 pupils in grade 4 (Finland and Sweden) and grade 5 (Iceland and Norway) from 62 different classes. Of these, 842 (69%) parents consented to participate. However, during the study, five pupils dropped out (one each in Iceland and Norway, and three in Sweden), leaving 837 (99% of those who consented to participate) pupils to participate throughout the whole study (Finland *n*=206, Iceland *n*=224, Norway *n*=210, and Sweden *n*=197). All included schools were run by the municipality and four of the five included towns were university towns with between 118,000 and 275,000 inhabitants at the time of the study.

In total, 85% of the participants’ parents/caregivers filled out the parent questionnaire (Table 1). Body weight and height was measured on 97% of the pupils. In total, 3,928 lunches were photographed, and for a majority of the pupils (78%), lunches for all 5 days were photographed. The short questionnaires before and after lunch were answered by 82 and 83% of the pupils, respectively, who had five complete days. The computer tests Stroop and CO-span were conducted for 93% of the pupils during the first study week. During 3 days in study week two, 46 randomly selected pupils from Sweden conducted Stroop and CO-span, and the computer test IVA+Plus was conducted in all countries by a mean of 169 randomly selected pupils per day (mean 25–68 pupils per country). A total of 37,413 observations of classroom learning behavior were performed.

**Table 1:** Collected data in the ProMeal-study between October 2013 and May 2014 in Finland, Iceland, Norway, and Sweden

	Finland (*n*=206)	Iceland (*n*=224)	Norway (*n*=210)	Sweden (*n*=197)	Total (*n*=837)
	
	*n* (%^a^)	*n* (%^a^)	*n* (%^a^)	*n* (%^a^)	*n* (%^a^)
Parent questionnaire	187 (91)	221 (99)	144 (69)	161 (82)	713 (85)
Body weight (kg)	200 (97)	224 (100)	209 (99)	179 (91)	812 (97)
Height (cm)	200 (97)	224 (100)	210 (100)	179 (91)	813 (97)
Body mass index (kg/m^2^)	200 (97)	224 (100)	209 (99)	179 (91)	812 (97)
School meal lunches					
Total lunches photographed	976 (95)	1,067 (95)	989 (94)	896 (91)	3,928 (94)
Number of pupils with 5 days photographed^b^	171 (83)	186 (83)	164 (78)	135 (69)	656 (78)
Questionnaire before lunch					
Total number of questionnaires filled out	966 (94)	1,072 (96)	1,005 (96)	919 (93)	3,962 (95)
Number of pupils with 5 days of filled out questionnaires	178 (86)	184 (82)	177 (84)	149 (76)	688 (82)
Questionnaire after lunch					
Total number of questionnaires filled out	976 (95)	1,056 (94)	991 (94)	912 (87)	3,935 (94)
Number of pupils with 5 days of filled out questionnaires	193 (94)	183 (82)	168 (80)	150 (76)	694 (83)
Observations in the classroom (number of observations in total)^c^	6,179 (60)	10,844 (97)	11,887 (113^d^)	8,503 (86)	37,413 (89)
Child Operation span and Stroop^e^					
Baseline measurement study week one	197 (96)	212 (95)	204 (97)	167 (85)	780 (93)
Measurements during study week 2 (only measured in Sweden)	–	–	–	46 (23)	46 (3)
The Integrated Visual and Auditory Continuous Performance Test (done by a randomized sub-sample of the pupils)^f^					
Test day 1	65 (32)	43 (19)	27 (13)	34 (17)	169 (20)
Test day 2	71 (34)	44 (20)	27 (13)	44 (22)	186 (22)
Test day 3	67 (33)	39 (17)	21 (10)	26 (13)	153 (18)
Empathy-based stories (total number of stories)					
Positive	95 (46)	103 (46)	104 (50)	86 (44)	388 (46)
Negative	95 (46)	100 (45)	85 (40)	85 (43)	365 (44)
Focus groups (number of groups, 5–8 pupils in each group)					
Gender-based divide	6 (3)	18 (8)	16 (8)	16 (8)	56 (7)
Mixed gender	13 (6)	0 (0)	9 (4)	0 (0)	22 (3)
School meal environment (number of schools observed)	9 (100)	6 (100)	6 (100)	9 (100)	30 (100)
Learning environment (number of schools observed)	9 (100)	6 (100)	6 (100)	9 (100)	30 (100)
School food organization^g^					
Level 1 (only measured in Sweden)	–	–	–	6 (100)	6 (100)
Level 2	9 (100)	6 (100)	6 (100)	6 (67)	27 (90)
Level 3	9 (100)	6 (100)	–	5 (56)	20 (67)

a The percentage of the total number of possible measurements of the participating pupils.

b The reason for less than 5 days were that the pupils either were absent from school one or more days, or that one or more pictures were missing in the series of photographs.

c Observations were made according to Blatchford et al. (33, (34)).

d In Norway, more observations than planned were made on each pupil and, consequently, the percentage exceeds 100%.

e Child Operation span (25) and Stroop test (26).

f The Integrated Visual and Auditory Continuous Performance Test (31).

g School food organization was studied with the e-tool School Food Sweden (35) in Sweden and adapted versions in the other countries.

Empathy-based stories were written by 90% of the participating pupils and focus groups were performed in 56 gender-based divided groups and 22 mixed gender groups.

### Participants

There was no difference in the gender distribution between the countries (*P=*0.472) (Table 2). Eighty-five percent of those filling out the parent/caregiver questionnaire were mothers/stepmothers. Pupils had a mean age of 10.5±0.36 years. The Finnish pupils were older than the Norwegian and Swedish pupils (10.7±0.34 years old in Finland versus 10.5±0.35 years old in Norway (*P*<0.001) and 10.6±0.33 years old in Sweden (*P*=0.002)). The mean body weight and height of the children was 38.3±7.70 kg and 145±6.80 cm. Mean body mass index (BMI) was 18.1±2.71 kg/m^2^. There was a difference in both body weight and BMI between pupils in Finland and Norway where the Norwegian pupils had lower body weight (37.2±6.60 kg versus 39.2±8.50 kg, *P*=0.033) and BMI (17.7±2.20 kg/m^2^ versus 18.5±3.20 kg/m^2^, *P*=0.020) than the Finnish pupils. The effect size for both differences was, however, low. The majority (81%) of the pupils were classified as normal weight, 16% overweight, and 3% as obese. There was a difference in the proportion of pupils classified as normal weight, overweight, and obese between countries. Finland had the highest proportion of pupils with obesity (6.5% versus 3%, 0.5%, and 3% in Iceland, Norway, and Sweden, respectively) (*P*=0.010). Iceland, on the contrary, had the highest proportion of children with overweight (18% versus 17%, 16%, and 12% in Finland, Norway, and Sweden, respectively) (*P*=0.010). A majority of the children from Finland, Iceland, and Sweden were born in the respective country (no data on Norway available). Iceland and Sweden had a higher proportion of non-native children than Finland (*P*=0.028). A somewhat higher proportion of children lived with parents/caregivers all of the time in Iceland and Sweden compared with Finland (*P*<0.001). In all countries, it was most common that the parents/caregivers had a university degree (*P*<0.001). There were no differences between countries in parents/caregivers reporting that their child had a diet-related disease or a chronic disease. A larger proportion of parents/caregivers rated their child's health as good (meaning, 8 to 10 on a VAS with 10 being the best possible condition) in Finland (98%) compared with Iceland (94%) and Sweden (89%) (*P*=0.002).

**Table 2:** Background information about pupils and parents/caregivers participating in the ProMeal-study between October 2013 and May 2014 in Finland, Iceland, Norway, and Sweden

	Finland (*n*=187)		Iceland (*n*=221)		Norway (*n*=144)		Sweden (*n*=161)		
					
	Mean	SD	Mean	SD	Mean	SD	Mean	SD	*P*^a^
Age (years)^a^	10.7	0.3	10.6	0.3	10.5	0.3	10.6	0.3	**<0.001**
Body weight (kg)^b^	39.2	8.5	38.9	8.1	37.2	6.6	37.9	7.2	**0.022**
Height (cm)^c^	145	6.8	146	6.8	145	6.9	145	6.5	0.463
BMI (kg/m^2^)^d^	18.5	3.2	18.3	2.8	17.7	2.2	17.9	2.6	**0.016**
	%		%		%		%		
			
Sex, girls^e^	52.0		49.0		56.0		51.0		0.472
Non-native pupils	2.0		7.0		–		5.0		**0.028**
Child live with both parents all the time	62.0		89.0		–		90.0		**<0.001**
Education parent 1^f^									
≥10–12 y	38.5		32.0		27.0		34.0		
University degree	53.5		58.0		73.0		64.0		**<0.001**
Other/none	8.0		10.0		0.0		1.0		
Employed parent one (or other occupation)^f^	89.0		87.0		–		91.0		0.488
Body mass index classification									
Overweight	16.5		18.0		16.0		12.0		**0.010**
Obesity	6.5		3.0		0.5		3.0		
Child has a diet-related disease	13.0		7.0		11.0		6.0		0.067
Child has other chronic disease	17.0		15.0		11.0		9.0		0.123
Parents who estimate the child's health as good^g^	98.0		94.0		–		89.0		**0.002**

The difference between the groups were compared with a Chi-Square test on categorical variables (the Fishers exact test was used for the variables in which the parents rate the child's health and BMI classification). One-way between-groups ANOVA with *post-hoc* tests was used to compare means of continuous variables. (In the present table, only *P*-values from the ANOVA are presented. *P*-values from *post-hoc* tests are presented in the text.)

a Finland *n*=206, Iceland *n*=224, Norway *n*=208, and Sweden *n*=190.

b Finland *n*=200, Iceland *n*=224, Norway *n*=209, and Sweden *n*=179.

c Finland *n*=200, Iceland *n*=224, Norway *n*=210, and Sweden *n*=179.

d Body mass index: Finland *n*=200, Iceland *n*=224, Norway *n*=209, and Sweden *n*=1,879.

e Finland *n*=206, Iceland *n*=224, Norway *n*=210, and Sweden *n*=197.

f Education degree and employment for the parent who filled out the questionnaire.

g Parents were asked to rate their child's health on a visual analogue scale, 1–10. In the present paper, 8–10 on the VAS scale is defined as good health.

Bold P-values indicate a statistically significant difference between countries.

## Discussion

The present study is unique with its Nordic approach in studying the effect of school lunch on overall healthiness of diet, cognitive function, classroom learning behavior, and pupils’ own understandings and experiences of school lunch. The Nordic countries have been proposed as a global health lab (36), given their many similarities in, for example, culture, dietary habits, and diet-related diseases. The present study has used these similarities as a base for studying different aspects of school meals in the Nordic countries.

An overall strength with the present study is the large number of participating children. We aimed to recruit at least 200 pupils in each country and that goal was reached. The drop-out rate was low in all countries. Some of the children said they dropped out because of the strain of photographing the school lunch. The large number of participants made statistical comparisons more reliable and results more representative. Another unique aspect of the study is the multidisciplinary approach, such that different disciplines, in this case, food and nutrition, education, and psychology, met and studied a common research aim. To our knowledge, no other similar size studies have done this.

Cognitive function was tested through objective measures and will be evaluated in relation to dietary intake during the school lunch. Systematic observations of classroom learning behavior were also performed in order to be evaluated in relation to school lunch intake. To date, only a few studies have focused on the relationship between school lunch and cognitive function or classroom learning behavior in relation to lunch intake (5, 6, 33). A study situated in relatively poor areas in England showed substantial improvements in the national Key Stage 2 tests (literacy and science), as well as decreased absenteeism in schools as a result of the Jamie Oliver Campaign to improve the food in English schools (37). Improvement in children's classroom learning behavior has been shown in other intervention studies with an improved school lunch and eating environment (3, 4). One reason so few studies have been done in this area may be the methodological challenges of measuring dietary intake, cognitive function, and learning behavior. There are also many confounders involved that complicate the evaluation of cognitive function. Confounders that may affect a child's cognitive performance are sleep duration, physical activity, previous meals, physical or pedagogical environments, as well as social background, to name a few. In the present study, we tried to take into account as many of these confounders as possible because they may help explain some variations.

For future interventions aiming to improve children's food habits through school lunch, it is vital to know more about pupils’ own experiences and understandings of the school meal. This area has been little studied, and the present study will, to our knowledge, be the first to explore this important topic using qualitative methodology among a large sample of Nordic pupils. The two different methods used, focus group discussions and empathy-based stories, are expected to complement each other. In the focus groups, children are constructing understanding within groups (38), whereas empathy-based stories give children space to express their experiences and perspectives individually (23).

The participating pupils had a mean BMI within what is considered as normal weight according to the definition by Cole et al. (16). The proportion of overweight children in ProMeal was somewhat lower in Finland and Iceland and substantially lower in Sweden compared with what earlier studies have shown regarding the prevalence of overweight in these countries (39–41). In Norway, the prevalence of overweight was, in contrast, slightly higher than what earlier studies have reported (42). The proportion of children with obesity was lower in ProMeal than what earlier studies have reported in Iceland, Norway, and Sweden (40–42). In Finland, the obesity rate among the participating pupils was much higher than what has been reported before (39). The differences from earlier studies may be due to the relatively few pupils from each country compared with previous studies. The statistically significant difference of 0.8 kg/m^2^ in BMI between Finland and Norway had a low effect size according to Cohen's classification, which means that it is of little practical relevance. The Swedish parents/caregivers rated their children's health as somewhat lower compared to the other countries. Nevertheless, there was no statistically significant difference between countries as to how parents responded regarding whether their child had a diet-related disease or a chronic disease. The meaning parents/caregivers put into the word ‘health’ may vary within and between countries, which must be kept in mind when questions about health are interpreted.

The participation rate varied in the participating classes while Finland and Norway had the lowest participation rate in the included classes. A low participation rate increases the risk of selection bias, and there is a possibility that those who chose not to participate differ from the ones who chose to participate. A limitation is that it is not possible to study whether the groups differ since we do not have any information about those who declined participation. The participation rates in the parent/caregiver questionnaire were relatively good in Finland, Iceland, and Sweden (99, 91, and 82%) but lower than desired in Norway (69%) in spite of reminders through e-mails and phone calls. The background data for those not filling out the questionnaire are not known, but an analysis showed that the parents who did not answer the questionnaire were fairly evenly distributed among the participating schools. This is positive since the schools were situated in different socioeconomic areas. A further limitation is that some background questions from the parent/caregiver questionnaire were not included in the Norwegian questionnaire. This unfortunately, made comparisons of some background variables between all countries impossible. Missing data in the measurements carried out at school are due to, for example, ill children not present in school or practical difficulties in collecting data if the pupils were not at the same place at the same time. At many measuring occasions, pupils from one class were spread out in the school, for example, in smaller groups or individual pupils left the class for special teaching. Efforts were made to locate children not present in the classroom during measurements but not always successfully.

In spite of the schools being situated in different socioeconomic areas, the study population was homogeneous from a socioeconomic perspective because most of the parents/caregivers were highly educated and employed. A majority of the children lived continuously with parents/caregivers all the time, and a low proportion of children were non-native. Iceland and Sweden had a somewhat higher proportion of non-native pupils, which may add important information to the group as a whole. Efforts were made to recruit pupils from areas with different socioeconomic backgrounds, but not completely successfully. Because of the high inclusion of university towns and, consequently, the high education levels, the variation in socioeconomic status within each town was limited. Schools in areas with lower socioeconomic status were more difficult to recruit because the teachers thought that they already had enough to deal with and did not think they had the time to participate in a study. Because of the high socioeconomic status among the study population, the results must be interpreted with caution in relation to different socioeconomic contexts. From a methodological point of view, the homogenous groups can be an advantage, for example, when measuring the relationship between school lunch intake and cognitive function.

Dietary intake from lunches was assessed by a photographic method, which has been validated on the pupils in ProMeal (unpublished observations). The method worked well for the dietary assessment of pupils in the often time-limited and stressful school meal environment. Furthermore, the method is practical since it does not interfere much with the daily routine of the personnel and children, which is important when performing research involving the school setting. One downside of the method was missing photographs of taken left over food, for example, because pupils forgot to have their plates photographed, showing either what was taken or thrown away. In these instances, it was not possible to assess the child's total dietary intake from that particular lunch.

## Conclusion

ProMeal is, to date, the largest study done with the aim of studying Nordic pupils’ school lunch intake, total diet, cognitive function, learning behavior, and pupils’ own perspectives of school meals. The results of the study will give new insights into what future interventions need to focus on to improve pupils' dietary intake and learning. The study will also provide valuable information for policy making, not least in countries where the history of school meals is shorter than in some of the Nordic countries.
